# Predicting optical coherence tomography-derived diabetic macular edema grades from fundus photographs using deep learning

**DOI:** 10.1038/s41467-019-13922-8

**Published:** 2020-01-08

**Authors:** Avinash V. Varadarajan, Pinal Bavishi, Paisan Ruamviboonsuk, Peranut Chotcomwongse, Subhashini Venugopalan, Arunachalam Narayanaswamy, Jorge Cuadros, Kuniyoshi Kanai, George Bresnick, Mongkol Tadarati, Sukhum Silpa-archa, Jirawut Limwattanayingyong, Variya Nganthavee, Joseph R. Ledsam, Pearse A. Keane, Greg S. Corrado, Lily Peng, Dale R. Webster

**Affiliations:** 1 Google Health, Google, Mountain View, CA USA; 2 Faculty of Medicine, Department of Ophthalmology, Rajavithi Hospital, College of Medicine, Rangsit University, 2, Phayathai Road, Ratchathewi District, Bangkok, 10400 Thailand; 3 Google Research, Google, Mountain View, CA, USA; 4 EyePACS LLC, Santa Cruz, CA USA; 5 Meredith Morgan Eye Center, University of California, 200 Minor Hall, Berkeley, CA 94720-2020 USA; 6 Deepmind, London, UK; 7 NIHR Biomedical Research Centre for Ophthalmology, Moorfields Eye Hospital NHS Foundation Trust and UCL Institute of Ophthalmology, 2/12 Wolfson Building, 11-43 Bath Street, London, EC1V 9EL UK

**Keywords:** Diabetes, Biomedical engineering, Developing world

## Abstract

Center-involved diabetic macular edema (ci-DME) is a major cause of vision loss. Although the gold standard for diagnosis involves 3D imaging, 2D imaging by fundus photography is usually used in screening settings, resulting in high false-positive and false-negative calls. To address this, we train a deep learning model to predict ci-DME from fundus photographs, with an ROC–AUC of 0.89 (95% CI: 0.87–0.91), corresponding to 85% sensitivity at 80% specificity. In comparison, retinal specialists have similar sensitivities (82–85%), but only half the specificity (45–50%, *p* < 0.001). Our model can also detect the presence of intraretinal fluid (AUC: 0.81; 95% CI: 0.81–0.86) and subretinal fluid (AUC 0.88; 95% CI: 0.85–0.91). Using deep learning to make predictions via simple 2D images without sophisticated 3D-imaging equipment and with better than specialist performance, has broad relevance to many other applications in medical imaging.

## Abstract (web summary)

Diabetic eye disease is a cause of preventable blindness and accurate and timely referral of patients with diabetic macular edema is important to start treatment. Here the authors present a deep learning model that can predict the presence of diabetic macular edema from color fundus photographs with superior specificity and positive predictive value compared to retinal specialists.

## Introduction

Diabetic macular edema (DME) is a late stage of diabetic eye disease that is characterized by retinal thickening in the macula, often accompanied by hard exudate deposition, and resultant vision loss. It is one of the most common reasons for referrals to diabetic eye clinics and affects 3–33% of patients with diabetes^1^. The wide range of prevalences reflects the varied bases for defining the condition and the varied composition of the populations studied. Currently, the first-line treatment for DME is anti-vascular endothelial growth factor (anti-VEGF) agents^2–4^. To determine eligibility for anti-VEGF treatment of DME, most of the major clinical trials measured macular thickening using optical coherence tomography (OCT) and initiated treatment if a patient met the criteria for a particular type of DME^5,6^. This type of DME is now commonly called center-involved DME (ci-DME) in clinical practice. As such, findings on OCT along with impaired visual acuity has become a widely accepted standard of care for determining DME treatment^7^.

However, despite improvements in therapy, the detection of ci-DME remains a challenge, because adding OCTs to the screening process is too costly and logistically difficult to implement widely. Globally, there are 425 million patients with diabetes^8^ and most clinical guidelines recommend that all of them are screened annually^9^. Currently, selection of patients who may meet treatment criteria is performed during these screenings, which typically utilize monoscopic fundus images. These images are then evaluated for the presence of hard exudates within one optic disc diameter of the center of the macula, a proxy for ci-DME^10^. However, this proxy was developed based on an older standard of care and some studies have shown that hard exudates have both poor positive predictive value and poor sensitivity for ci-DME. MacKenzie et al.^11^ reported that only 42% of patients with hard exudates were found to have DME on OCT and Wang et al.^12^ reported that a third of patient eyes with DME detected on OCTs lacked features such as hard exudates on monoscopic fundus photographs. Wong et al.^13^ reported a false-positive rate of 86.6% for DME screening with existing strategies. As such, the potential of Diabetic Retinopathy (DR) screening and timely referral for DME is handicapped by an inability to reliably detect ci-DME via human evaluation of fundus photographs alone.

A potential solution lies in the use of deep-learning algorithms, which have been applied to a variety of medical image classification tasks^14–18^, including for retinal imaging^19–22^. Encouragingly, in addition to achieving expert-level performance for grading fundus images, deep-learning algorithms are able to make predictions for which the underlying association with fundus images were previously unknown, such as cardiovascular risk factors^23^ and refractive error^24^.

We hypothesized that deep learning could be leveraged to directly predict the OCT-derived ci-DME grade using monoscopic fundus photographs. In this study we show that ci-DME can be predicted with significantly higher specificity at the same sensitivity as doctors, in two independent datasets, using deep learning. In addition, the model also predicts the presence of intraretinal and subretinal fluid, which are clinically relevant tasks. Subsampling experiments show a likely increase in accuracy when training includes additional data. Training on cropped images of increasing sizes around the fovea and optic disc demonstrate that the model is largely informed by the area around the fovea.

## Results

### Deep learning can predict OCT features from fundus photographs

**Fig. 1:**
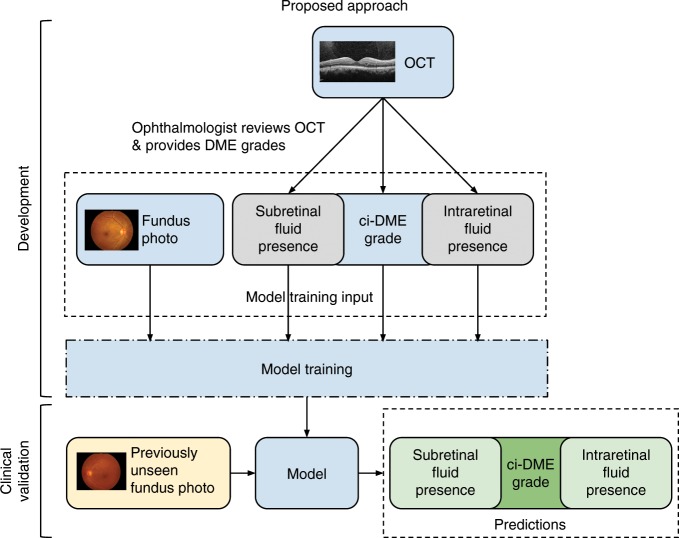
Illustration of our proposed approach for developing a ci-DME model. Ground truth for ci-DME were derived from a human grader analyzing the OCT for each case. In addition, subretinal fluid and intraretinal fluid presence grades were also collected. These ground truth labels and corresponding color fundus photos were used for model training. For clinical validation, the trained model takes in a new fundus photo and generates a predicted ci-DME grade, predicted subretinal fluid, and intraretinal fluid presence grades.

To leverage deep learning as a potential solution to reliably detect ci-DME, we propose developing a model trained on fundus photographs, but using ci-DME diagnoses derived from expert inspection of OCT as labels (Fig. 1). To train and validate the model, cases were gathered retrospectively from the Rajavithi Hospital in Bangkok, Thailand. As these cases were gathered from those referred into the retina clinic for further evaluation, the disease distribution is consistent with a population presenting to specialty clinics and are enriched for more severe disease as compared with a DR screening population. Details of the development and clinical validation datasets are presented in Table 1. The development dataset consisted of 6039 images from 4035 patients and the primary clinical validation dataset consisted of 1033 images from 697 patients. For some patients, only one eye was included, because the fellow eye fell under the exclusion criteria. ci-DME was conservatively defined as center point thickness ≥250 μm measured via manual caliper measurements excluding the retinal pigment epithelium^25,26^. We trained a model using this development dataset to predict ci-DME using fundus photographs as input.

**Table 1:** Baseline characteristics of the development and clinical validation datasets.

Characteristics	Development set	Primary clinical validation set	Secondary clinical validation set
Dataset	Thailand dataset	Thailand dataset	EyePACS-DME dataset
Number of patients	4035	697	554
Number of fundus images	6039	1033	990
Camera used for fundus images	Kowa VX-10	Kowa VX-10	Canon CR-DGi
OCT device used for determining ci-DME	Heidelberg Spectralis	Heidelberg Spectralis	Optovue iVue
Age: mean, years (SD)	55.6 (10.8) *n* = 6038	55.8 (10.8) *n* = 1033	62.0 (9.8) *n* = 990
Gender (% male)	60.8% *n* = 6036	62.4% *n* = 1031	50.1% *n* = 990
Central retinal thickness: mean, μm (SD)	263.8 (146.5) *n* = 6039	258.4 (132.8) *n* = 1033	254.4 (56.3) *n* = 990
ci-DME, Center Point Thickness ≥ 250 μm in Thailand dataset. Central Subfield Thickness ≥ 300 μm in the Eyepacs-DME dataset	28.3% *n* = 6039	27.2% *n* = 1033	7.8% *n* = 990
Subretinal fluid presence	15.7% *n* = 6039	15.1% *n* = 1033	NA
Intraretinal fluid presence	45.5% *n* = 6039	46.3% *n* = 1033	NA

It is noteworthy that the difference between total *n* and subcategories is missing data (e.g., not all images had age or sex)

### Model predicts OCT-based DME features better than retinal specialists

**Fig. 2:**
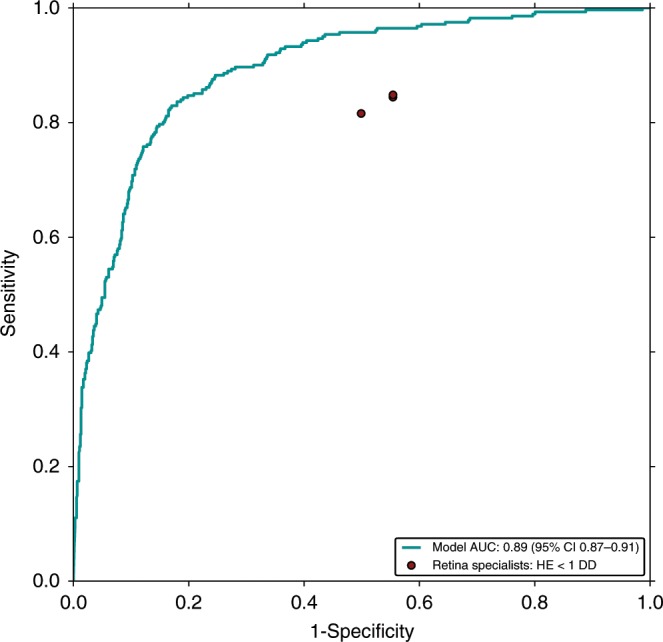
Receiver operating characteristic curve of the model compared to retinal specialists. The retinal specialists’ grades for predicting ci-DME on the primary clinical validation set are shown as red dots. All methods (i.e., the model and retinal specialists) rendered their grades using monoscopic fundus images only. The ground truth for ci-DME was derived using OCT (center point thickness ≥ 250 μm).

Our model showed a higher performance in detecting cases with and without ci-DME from monoscopic fundus images compared with manual grading of fundus images (Table 2 and Fig. 2). For ci-DME, the model had a sensitivity of 85% at a specificity of 80%. Three retinal specialists had sensitivities ranging from 82% to 85% at specificities ranging from 45% to 50% (Supplementary Table 1). The performance improvements held true even if other common criteria for calling DME for monoscopic images were used (Supplementary Fig. 1), such as changing the definition of DME based on the location of the hard exudates. Additional analyses were also performed at other thickness thresholds for ci-DME at center point thickness ≥ 280 μm and ≥ 300 μm, which showed similar or better results compared with the conservative ≥ 250 μm cutoff point without model retraining (Supplementary Fig. 2). When compared with manual grading, our model had a 30–35% absolute higher specificity at the same sensitivity (*p* < 0.001 for comparison with each retinal specialist). When matched to have the same specificity, the model had a 11–14% absolute higher sensitivity (96% vs. 82–85%, *p* < 0.001 for all comparisons).

**Table 2:** Performance metrics of the model and retinal specialists on the primary clinical validation set.

Metric	Model	Specialist 1	Specialist 2	Specialist 3
Positive predictive value (%), 95% CI	61% [56–66%] *n* = 1033	37% [33–40%] *n* = 1004	36% [33–40%] *n* = 987	38% [34–42%] *n* = 1001
Negative predictive value (%), 95% CI	93% [91–95%] *n* = 1033	88% [85–91%] *n* = 1004	89% [85–92%] *n* = 987	88% [84–91%] *n* = 1001
Sensitivity (%), 95% CI	85% [80–89%] *n* = 1033	84% [80–89%] *n* = 1004	85% [80–89%] *n* = 987	82% [77–86%] *n* = 1001
Specificity (%), 95% CI	80% [77–82%] *n* = 1033	45% [41–48%] *n* = 1004	45% [41–48%] *n* = 987	50% [47–54%] *n* = 1001
Accuracy (%), 95% CI	81% [79–83%] *n* = 1033	56% [52–59%] *n* = 1004	56% [52–59%] *n* = 987	59% [56–62%] *n* = 1001
Cohen’s Kappa, 95% CI	0.57 [0.52–0.62] *n* = 1033	0.21 [0.16–0.25] *n* = 1004	0.21 [0.16–0.25] *n* = 987	0.24 [0.19–0.28] *n* = 1001

For the model we chose an operating point that matched the sensitivity of the retinal specialists to calculate the metrics. The performance metrics for the model were calculated on the entire primary clinical validation set; for the retinal specialists it was calculated only on the images that they marked as gradable. Brackets denote 95% confidence intervals. *n* = number of images

**Fig. 3:**
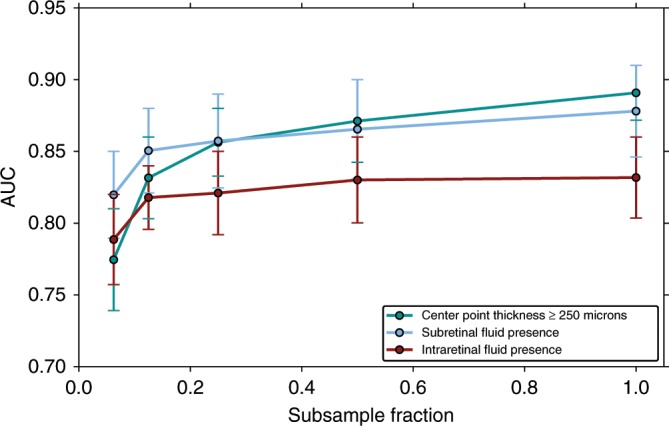
Effect of data size on prediction performance on the primary clinical validation set. A subsampled fraction of 1.0 indicates the entire training dataset. Model performance continues to increase with increased data, suggesting that the accuracy of predicting ci-DME, subretinal fluid, and intraretinal fluid presence will likely improve if the model is trained with more data. Error bars are 95% confidence interval using *n* = 2000 samples with replacement.

In addition to predicting ci-DME, our model was able to predict presence of intraretinal and subretinal fluid. Our model had an area under the curve (AUC) of 0.81 (95% confidence interval (CI): 0.81–0.86) for detecting intraretinal fluid presence and an AUC of 0.88 (95% CI: 0.85–0.91) for subretinal fluid presence (Fig. 3).

### Model generalizes to a secondary validation set

**Fig. 4:**
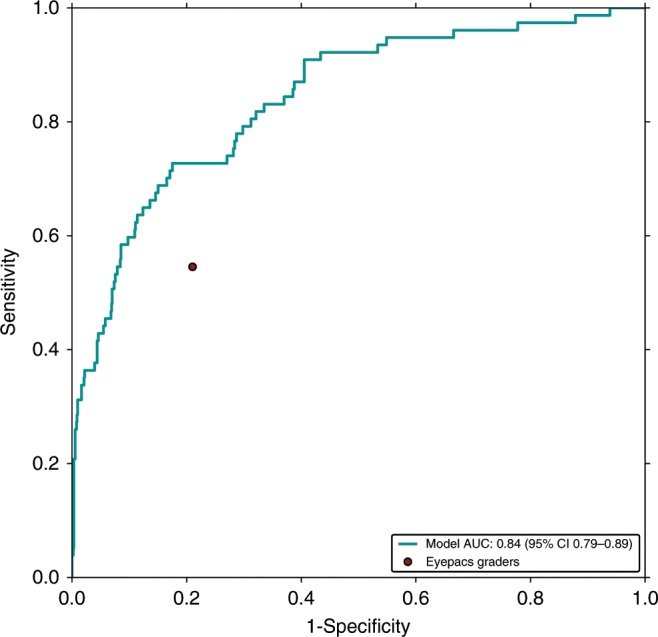
Receiver operating characteristic curve of the model compared with eyepacs graders. The graders’ grades for predicting ci-DME on the secondary clinical validation set are shown as a red dots. All methods (i.e., the model and eyepacs graders) rendered their grades using monoscopic fundus images only. The ground truth for ci-DME was derived using OCT (central subfield thickness ≥ 300 μm).

In addition to the primary clinical validation dataset, the model was also applied to a secondary validation dataset, EyePACS-DME, to examine the model’s generalizability. This dataset consists of 990 images with moderate, severe non-proliferative DR or proliferative DR, a subset of data previously gathered during another DME study^27^. The images were gathered using a Canon CR-DGi camera and OCTs were taken with a Optovue iVue machine from a US-based population (see Methods). There are some notable differences in this dataset in comparison with the primary validation dataset, particularly in terms of defining and measuring ci-DME based on central subfield thickness and incorporation of inclusion/exclusion criteria (Supplementary Table 2). Based on this different definition and inclusion criteria, the number of ci-DME cases in the secondary validation set was 7.8% compared with 27.2% in the primary clinical validation set. Thus, the model performance on the datasets cannot be compared directly in terms of absolute values (especially for metrics such as positive predictive value (PPV), which depend a lot on the priori distribution). However, relative comparisons between the model and graders (in this instance EyePACS certified graders) can be drawn (Fig. 4 and Table 3). Similar to the results of the primary validation, our model had a PPV roughly twice that of manual grading using hard exudates as proxy (35% [95% CI: 27–44%] vs. 18% [95% CI: 13–23%]) and similar negative predictive value (NPV) (96% [95% CI: 95–98%] vs. 95% [95% CI: 94–97%]). This translated to a similar sensitivity (57% [95% CI: 47–69%] vs. 55% [43–66%]) but higher specificity (91% [95% CI: 89–93%] vs. 79% [95% CI: 76–82%]).

**Table 3:** Performance metrics of the model and eyepacs graders on the secondary clinical validation set.

Metric	Model	EyePACS Graders
Positive predictive value (%), 95% CI	35% [27–44%]	18% [13–23%]
Negative predictive value (%), 95% CI	96% [95–98%]	95% [94–97%]
Sensitivity (%), 95% CI	57% [47–69%]	55% [43–66%]
Specificity (%), 95% CI	91% [89–93%]	79% [76–82%]
Accuracy (%), 95% CI	88% [86–91%]	77% [74–80%]
Cohen’s Kappa, 95% CI	0.38 [0.29–0.47]	0.17 [0.11–0.24]

For the model we chose an operating point that matched the sensitivity of the eyepacs graders to calculate the metrics. Brackets denote 95% confidence intervals. *n* = 990 images for all calculations

### More data leads to better model performance

Sub sampling experiments, where new models were trained using titrated fractions of the dataset, showed that model performance continued to increase with larger training sets (see Fig. 3—where AUC increases with sample size). These results suggest that the accuracy of this prediction will likely continue to increase with dataset sizes larger than that in this study.

### Features around the fovea are most relevant

**Fig. 5:**
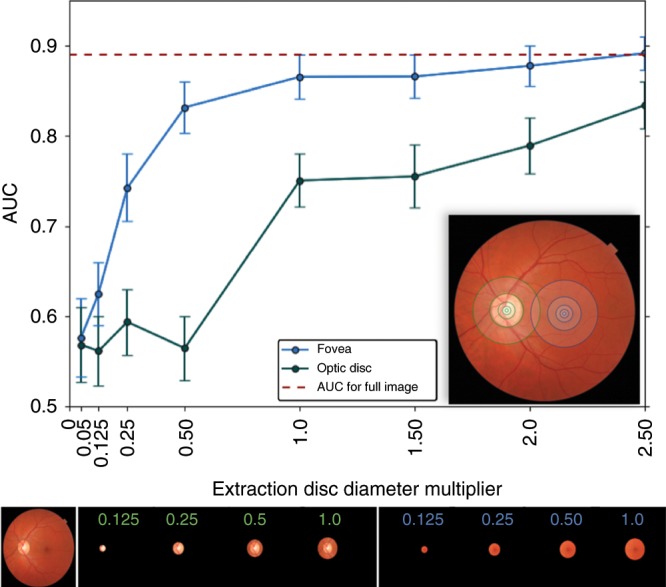
Model performance on crops around fovea and optic disk. The plot shows model performance in predicting ci-DME on the primary clinical validation set, as measured by AUC when cropped circular images are used to train and validate the model. The blue line indicates the performance when cropped circular images of different sizes (radius of multiples of disc diameter from 0.05 to 2.5) centered at the fovea are used; the green line indicates the corresponding performance when the crops are centered at the optic disc. (Inset) Image depicting some of the regions of different radii (0.05, 0.125, 0.25, 0.5, and 1 disc diameter) around the fovea and optic disc. Bottom panel: Fundus image, followed by a sample of the crops extracted by centering at the optic disc (green) and fovea (blue), with the extraction radius in multiples of disc diameter indicated in each crop. Error bars are 95% confidence interval using *n* = 2000 samples with replacement.

Figure 5 presents an analysis of the areas in the fundus image relevant for the model. When the model was trained on cropped fundus images containing only 0.25 optic disc diameter around the fovea (blue line), it achieved an AUC of 0.75. When it had access to 1.0 optic disc diameter around the fovea, the model achieved an AUC > 0.85, comparable with its performance on the full fundus image. However, the model trained on the region around the optic disc needed to see a lot more context (2.5 optic disc diameter) around the optic disc center to achieve an AUC exceeding 0.8. Based on these results, we believe the model primarily utilizes the area around the fovea to make ci-DME predictions.

## Discussion

Our previous work has shown that deep learning can be leveraged to make predictions from fundus photographs, such as cardiovascular risk factors and refractive error, which are not possible by human experts^23,24^. This study describes a model that far exceeds expert performance for such a prediction, but one that has high clinical relevance and potentially important implications for screening programs worldwide. The resultant model performed significantly better than retinal specialists for detecting ci-DME from fundus images in two datasets from very different populations. DME is the major cause of visual loss from DR. Prior to the use of anti-VEGF injections, the Early Treatment of Diabetic Retinopathy Study (ETDRS) showed that treatment of a subtype of DME with focal laser photocoagulation decreased the chance of vision loss^28^. Today, with anti-VEGF injections, the treatment of ci-DME can improve vision by ~10–13 letters as measured using the ETDRS visual acuity chart^5^. Anti-VEGF injections are now largely considered the gold standard of care with evidence that shows that delaying treatment of DME could lead to suboptimal visual improvement^29^. However, the current grading guidelines in screening programs were developed before the advent of anti-VEGF therapy and are not specifically designed for detecting ci-DME. The development of models that can better detect ci-DME in DR screening programs using existing equipment (color fundus cameras) is both scientifically interesting and clinically impactful.

For DR screening in particular, our model may lead to fewer false negatives for DME. Decreasing missed referrals for patients with ci-DME presenting with no hard exudates is a clear advantage of such a system. Visual acuity alone is not enough to rule out ci-DME, as baseline characteristics from some well-known cohorts suggest that a substantial percentage of eyes with ci-DME still have good vision^30,31^. In addition, decreasing false positives is also important in resource-constrained settings. Although many screening programs recommend closer follow-up for patients with mild or worse DR, the urgency of follow-up varies widely, especially in low resource settings. Per international guidelines (International Council of Ophthalmology Guidelines, American Academy of Ophthalmology), for patients with mild DR and no macular edema, referral is not always required and patients can be rescreened in 1–2 years in low/intermediate resource settings and 6–12 months in high resource settings. However, patients with suspected ci-DME need to be referred within a month. For patients with moderate non-proliferative DR and no macular edema, follow-up changes from 6 to 12 months in low/intermediate resource or from 3 to 6 months in high resource settings to 1 month (all resource settings) when there is ci-DME^32,33^. In this study, roughly 88% of the moderate non-proliferative patients from the EyePACS-DME dataset and 77% of those from the Thailand dataset, who would have been referred urgently (and unnecessarily) using a hard exudate-based referral criterion did not have ci-DME. Higher urgency referral of patients with moderate non-proliferative DR without DME (but presenting hard exudates) can be a major issue where there are limited resources for evaluation and treatment.

Furthermore, the center point thickness distribution of the false-positive and false-negative instances is better for the model when compared with retina specialists (Supplementary Fig. 3). For the model, 28% of the false positives have thickness >225 μm and 35% of the false negatives have thickness <275 μm. In comparison, for retina specialists, only 20% of the false positives have thickness >225 μm and 17% of the false negatives have thickness <275 μm. This shows that a significantly larger fraction of the model false positives and negatives are borderline cases as compared with retina specialists. In addition, the new model seems to be able to detect the presence of intraretinal and/or subretinal fluid, both of which merit closer monitoring and possibly treatment^34^. The ability to detect these pathologies is not a task that doctors can do accurately from fundus images.

Although the performance of the models on the secondary dataset is lower than that of the models on the primary dataset, the performance of the human graders on the secondary dataset is proportionally lower as well. From the primary to secondary dataset, PPV of the model decreased from 61% to 35%, whereas of the graders decreased from 37% to 18%; sensitivity of the model decreased from 85% to 57%, whereas of the graders decreased from 84% to 55%. However, NPV of the model increased from 93% to 96%, whereas of the graders increased from 88% to 95%; specificity of the model increased from 80% to 91%, whereas of the graders increased from 47% to 79%. These results reflect the inherent differences between the two datasets but still support the better performance of the model over graders on both datasets. Although the models trained in this study are more accurate than manual grading and the low PPVs are not inconsistent with what has been reported for other applications in the literature^35,36^, there is capacity for improvement. Given the results of the subsampling experiments, it is likely that the accuracy of the model may continue to increase with larger dataset sizes.

From a scientific point of view, this work demonstrates the potential of deep learning to enable diagnostics from inexpensive hardware, which was only previously possible from expensive equipment. It also lays the groundwork for understanding how the model makes these predictions. The explanation technique employed in this study indicated that the region around the fovea is more relevant than the region near the optic disc for DME prediction from fundus images. Future work could involve diving deeper into the features around this area that is picked up by deep learning but overlooked by retinal specialists.

In a small non-randomized study, Scott et al.^37^ showed a beneficial effect of focal and grid laser for eyes without central involvement that meet an older criteria for treatment known as clinically significant macular edema, similar to the initial ETDRS findings. These patients need to be referred from a DR screening program for closer follow-up. Our model does not evaluate such cases. To address this, one would include stereoscopic imaging in addition to OCT as ground truth to train model(s) to specifically identify these cases. Although there is some evidence of generalization to a secondary dataset, the confidence intervals are wide and the criteria for ci-DME for the EyePACS-DME dataset were different from those of the Thailand dataset. Some of the performance metrics reported in this study such as PPV and NPV are relevant only to populations whose severity distribution is similar to that of this study (e.g., patients referred to specialist clinics). Further studies should validate the model on additional larger datasets from other settings, including screening settings from other regions or geographies. Future studies should also include better standardization for ci-DME and inclusion/exclusion criteria, as well as sub-analysis of patients who were treated for DME. Moreover, additional data diversity such as the use of ci-DME labels derived from other OCT devices by other manufacturers should be included in future work. As the model was trained using treatment-naive fundus images, training on multiple images per eye (including with stereo pairs), and on eyes that have been treated for DME in the past could lead to better model performance. Although our cropping experiments (Fig. 5) show that the model looks at the region around the fovea for predicting ci-DME, future work could further explore interpretability of the model^37^. Lastly, future work could also include health economic analysis to study the cost-effectiveness of such an approach.

Nevertheless, this study demonstrates that deep learning can be leveraged to identify the presence of ci-DME using the cheaper and more widely available fundus photograph, at an accuracy exceeding that of manual grading using expert-derived rules. Similar approaches could be particularly valuable for other medical images, such as using radiographs or low-dose computed tomography to detect conditions that would otherwise require more expensive imaging techniques that expose patients to higher radiation doses. Importantly, we also use crops around the fovea and optic disc to explain how the model is making these predictions, lending confidence that the predictions will generalize to new unseen datasets.

## Methods

### Ethics approvals

This study was approved by the Ethics Committees or Institutional Review Boards of hospitals or health centers where retinal images of patients with diabetes were used in this study, including the Rajavithi Hospital (Bangkok, Thailand), Alameda Health Service (Alameda, CA, USA), and the University of California, Berkeley (Berkeley, CA, USA) in accordance with the Declaration of Helsinki. Patients gave informed consent allowing their retinal images to be used. This study was registered in the Thai Clinical Trials Registry, Registration Number TCTR20180818002.

### Datasets

For algorithm development, 7072 images were gathered retrospectively from diabetic patients presenting to the retina clinic at Rajavithi Hospital in Bangkok, Thailand, from January 2010 to February 2018. Only cases that were naive to treatment (both intravitreal injections and lasers) were included. Cases where macular lesions may have hyporeflective spaces on OCT, such as Macular Telangiectasia Type 2, may interfere with the diagnosis of DME, such as idiopathic epimacular membrane, macular edema from other causes, or proliferative DR with neovascular membrane affecting the macula, were excluded from analysis.

Retinal fundus images were obtained using Kowa color fundus camera (VX-10 model, Kowa, Aichi, Japan). A single macula-centered color fundus photograph per eye was used in the study. If available, imaging from both eyes were included. OCTs were obtained using the Heidelberg Spectralis OCT (Heidelberg Engineering GmbH, Germany) and thickness measurements were measured manually (see below for measurement procedures).

Of the 7072 images in the dataset, 6039 were used for development, whereas 1033 were set aside for clinical validation. All images from a patient was present in either in development or validation sets, but not both. Fundus photographs in the validation set were manually graded by US board-certified retinal specialists to assess the presence and location of hard exudates (yes, no, ungradable, within 500 μm or 1 disc diameter or 2 disc diameters from the center of the macula) and focal laser scars. In addition, retinal specialists provided their best clinical judgment of the presence of DME that took into account all the pathology present in the image.

To study generalizability of the model, the algorithm was applied to another dataset, EyePACS-DME, which is a subset of data that had been previously gathered for another DME study^27^. This dataset consisted of 990 macula-centered images from 554 patients with at least moderate DR based on grading by certified EyePACS graders (to roughly match the population of those who would be presenting to a retina clinic). No other exclusion criteria were applied to this dataset (e.g., exclusion of epiretinal membrane, etc). Fundus images were taken with a Canon CR-DGi camera (Ōta, Tokyo, Japan) and OCTs were taken with a Optovue iVue machine (Fremont, CA, USA).

### Measurement and assessment of OCT scans

For the Thailand dataset, central subfield thickness, the value representing the thickness of the center of the macula in clinical trials for DME^26^, was not available in all eyes in the developmental dataset; therefore, center point thickness, which was found to have high correlation with the central subfield thickness^25^, was measured for each eye to represent the thickness of the center of the macula.

The center point thickness of an eye of a patient was manually measured on the axis of the OCT scan where there was a slight elevation of the ellipsoid zone and the gap between the photoreceptor layer outer segment tip and the ellipsoid zone was the widest, indicating the center of the fovea where the cone cell density is the highest. Manual measurement was conducted using the straight-line measurement vector available with the Spectralis Eye Explorer software. The vector was put perpendicular to the highly reflective band of retinal pigment epithelium with one side of the vector rested on the highly reflective line of cone outer segment tip and the other side on the internal limiting membrane. Retinal pigment epithelium thickness was not included in this measurement. Intraretinal fluid was defined as present when a cystoid space of hypo-reflectivity was found within 500 μm of the foveal center of any OCT scans of a patient. Subretinal fluid was defined as present when a space of hypo-reflectivity was found between the retina and retinal pigment epithelium within 500 μm of the foveal center of any OCT scans of a patient.

The measurement of center point thickness and the assessment of presence of intraretinal fluid and subretinal fluid were conducted by two medical doctors experienced in clinical research and supervised by retinal specialists. Five percent of patients were randomly selected to confirm all three measurements by a retinal specialist with 20 years of post-certification experience.

Eyes were divided into cases of no ci-DME and ci-DME. ci-DME was conservatively defined as eyes with ≥250 μm center point thickness, excluding the retinal pigment epithelium based upon manual measurement^25,26^. In addition to ci-DME, we also trained the model in a multi-task fashion to predict subretinal fluid and intraretinal fluid (details below). Although cases with subretinal fluid and intraretinal fluid were not strictly included in the criteria in the clinical anti-VEGF trials for DME, referral for follow-up is warranted for these cases.

For the EyePACS-DME dataset, the manufacturer’s automated segmentation algorithm was used to measure central subfield thickness. A cutoff of 300 μm central subfield thickness was used as the cutoff point for ci-DME based on machine-specific adjustments^38^. The presence of intraretinal and subretinal fluid were not available in this dataset.

### Model

Our deep learning algorithm for predicting ci-DME was built using the methods described by Gulshan et al.^19^, using the Inception-v3^39^ neural network architecture. Briefly, we used a convolutional neural network^40^ to predict ci-DME (center point thickness ≥250 μm), subretinal fluid presence and intraretinal fluid presence in a multi-task manner. The input to the neural network was a color fundus photograph and the output was a real-valued number between 0 and 1 for each prediction, indicating its confidence. For other hyperparameter and training details see Supplementary Methods.

The parameters of the neural network were determined by training it on the fundus images and OCT-derived ci-DME grades in the development dataset. Repeatedly, the model was given a fundus image with a known output as determined by a grader looking at the patient’s corresponding OCT. The model predicted its confidence in the output, gradually adjusting its parameters over the course of the training process to become more accurate. Itis noteworthy that the model never sees the actual OCT image during training or validation.

### Evaluating the algorithm

To evaluate the performance of the model, we used the receiver operating characteristic (ROC) curve and calculated the AUC. The performance of the retinal specialists was marked by points on this curve, indicating their sensitivity and specificity (Fig. 2). The same model was also evaluated at increasing thresholds of thickness for ci-DME, without retraining (Supplementary Fig. 2). By choosing an operating point on the ROC curve that makes the model’s specificity match that of retinal specialists, we also evaluated the model using Sensitivity, Specificity, PPV, NPV, Accuracy, and Cohen’s Kappa score^41^ (Table 2).

### Statistical analysis

To assess the statistical significance of these results, we used the non-parametric bootstrap procedure: from the validation set of *N* images, sample *N* images with replacement and evaluate the model on this sample. By repeating this sampling and evaluation 2000 times, we obtain a distribution of the performance metric (e.g., AUC) and report the 2.5 and 97.5 percentiles as 95% confidence intervals. For statistical comparisons, the two-tailed paired permutation test was used with 2000 random permutations^42^.

### Model explanation

We performed two experiments to determine which regions in a fundus image are most informative of DME. We focussed on two regions, the macula and the optic disc. First, a group comprising ophthalmologists and optometrists manually marked the fovea and disc for all images in the Thailand dataset. We then trained and evaluated our model looking only at the region that is within a factor of optic disc diameters around the fovea (or equivalently the optic disc) with the rest of the fundus blacked out. We trained and evaluated different models for different radii, increasing the area that the model looks at to understand the importance of these regions in making the prediction (Fig. 5).

### Reporting summary

Further information on research design is available in the Nature Research Reporting Summary linked to this article.

## Supplementary information

Supplementary Information

Reporting Summary

## Data Availability

We make use of the machine learning framework TensorFlow (https://github.com/tensorflow/tensor-flow) along with the TensorFlow library Slim (https://github.com/google-research/tf-slim), which provides an implementation of the Inception-V3 architecture (https://github.com/google-research/tf-slim/blob/master/tf_slim/nets/inception_v3.py). Our experimental framework makes use of proprietary libraries and we are unable to publicly release this code. We detail the experiments and implementation details, including the details of data augmentation, model architecture, hyperparameters, and weights initialization used, in the Methods and Supplementary Information, to allow for independent replication.

## Source data

Source Data

## Data availability

Data are available from the corresponding author(s) upon reasonable request. Restrictions apply to the sharing of patient data that support the findings of this study. This data may be made available to qualified researchers upon ethical approvals from Rajavithi Hospital and EyePACS. The source data underlying Figs. 2–5, Tables 1 and 2, and Supplementary Figs. 1–3 are provided as a Source Data file.
