# Supplementary material for: Predicting optical coherence tomography-derived diabetic macular edema grades from fundus photographs using deep learning
Source: Nat Commun. 2020 Jan 8;11:130. doi: 10.1038/s41467-019-13922-8 (PMC6949287; doi:10.1038/s41467-019-13922-8)
Supplement: Supplementary file 1 — Supplementary Information [file 41467_2019_13922_MOESM1_ESM.pdf]

Predicting optical coherence tomography-derived diabetic macular edema grades from fundus photographs using deep learning

Varadarajan and Bavishi et al.

Supplementary Information

Supplementary Figures

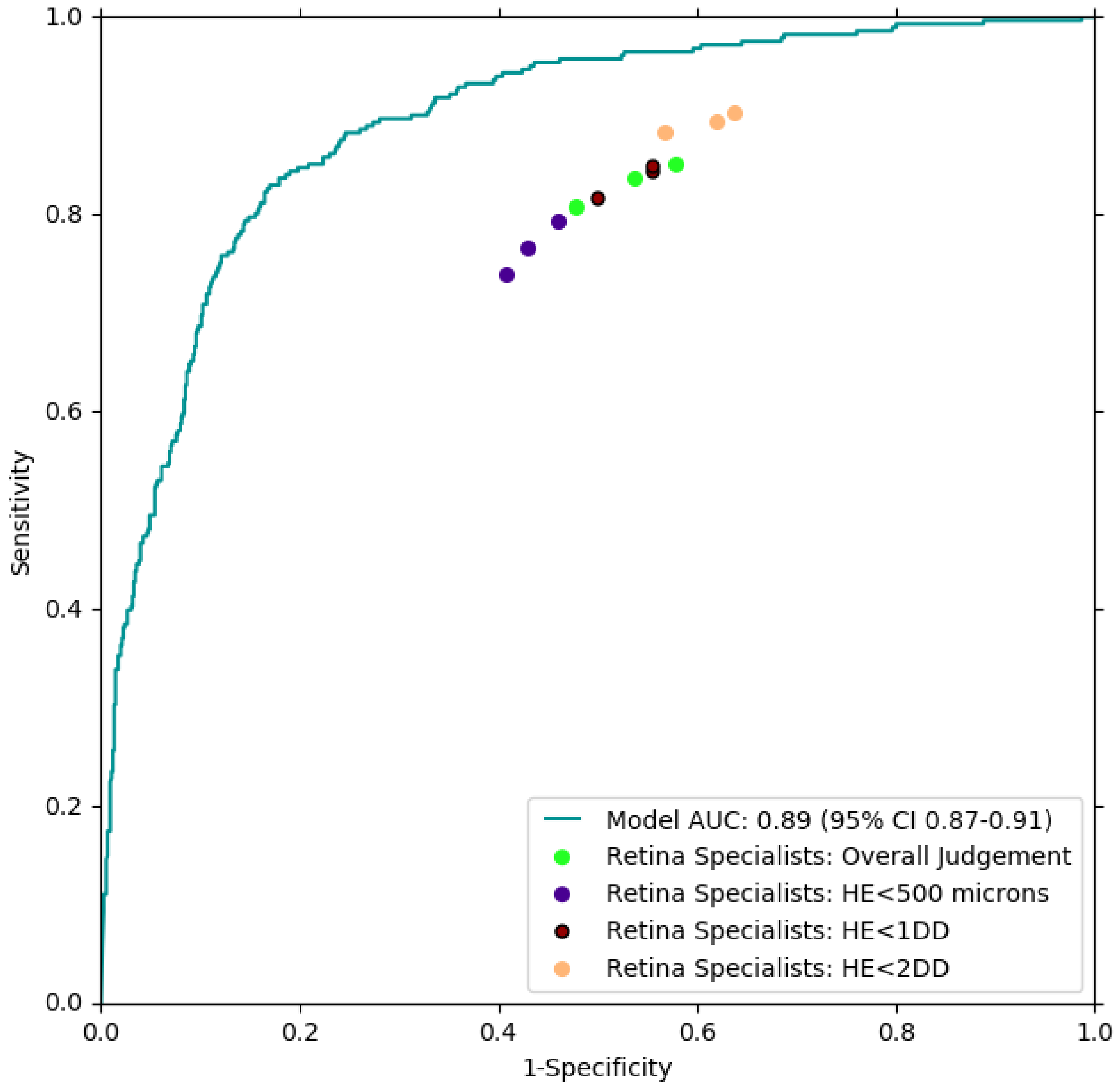

Supplementary Figure 1: Receiver operating characteristic curve of the model with retinal specialists' grades shown as red, yellow, and cyan dots for predicting ci-DME using different criteria for manual grading for DME on the primary clinical validation set. All methods (i.e. the model and retinal specialists) rendered their grades using monoscopic fundus images only. The ground truth was derived using OCT (center point thickness>=250 μm).

A. CPT>=280 μm

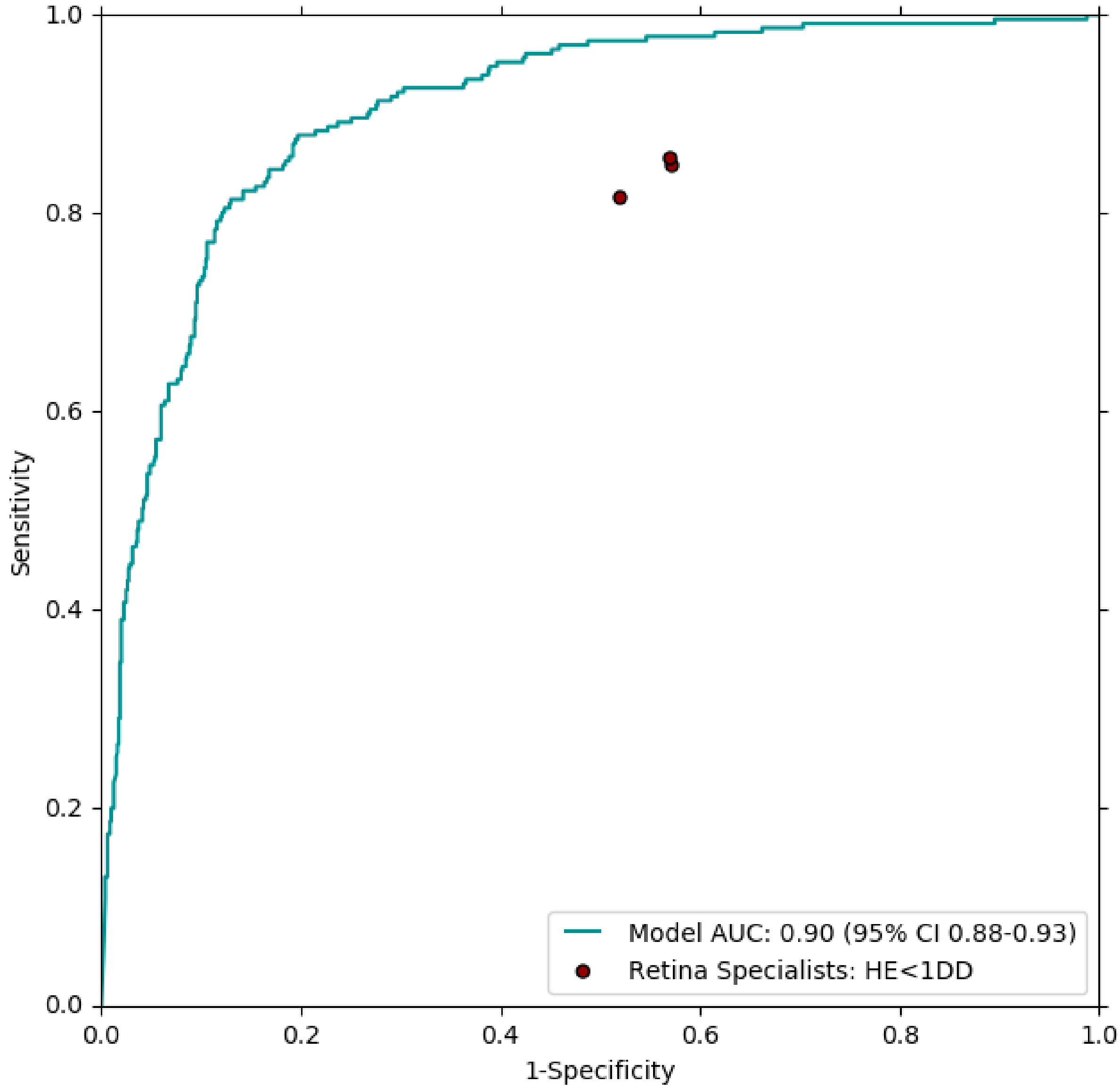

1.0
0.8
0.6
0.4
0.2
0.0
Sensitivity
0.0
0.2
0.4
0.6
0.8
1.0
1-Specificity
Model AUC: 0.90 (95% CI 0.88-0.93)
Retina Specialists: HE<1DD

B. CPT>=300 μm

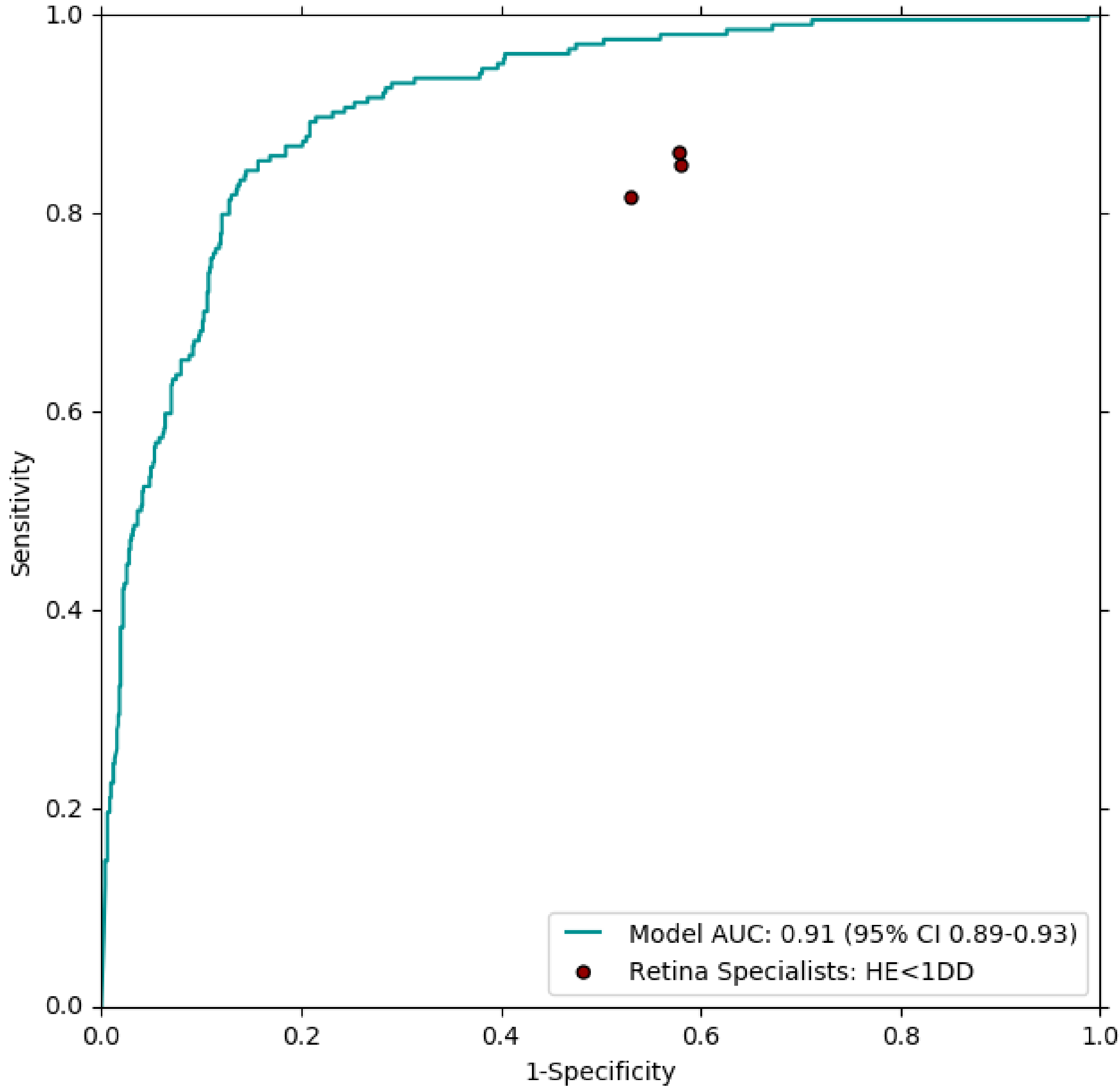

1.0
0.8
0.6
0.4
0.2
0.0
Sensitivity
0.0
0.2
0.4
0.6
0.8
1.0
1-Specificity
Model AUC: 0.91 (95% CI 0.89-0.93)
Retina Specialists: HE<1DD

C. CPT>=320 µm

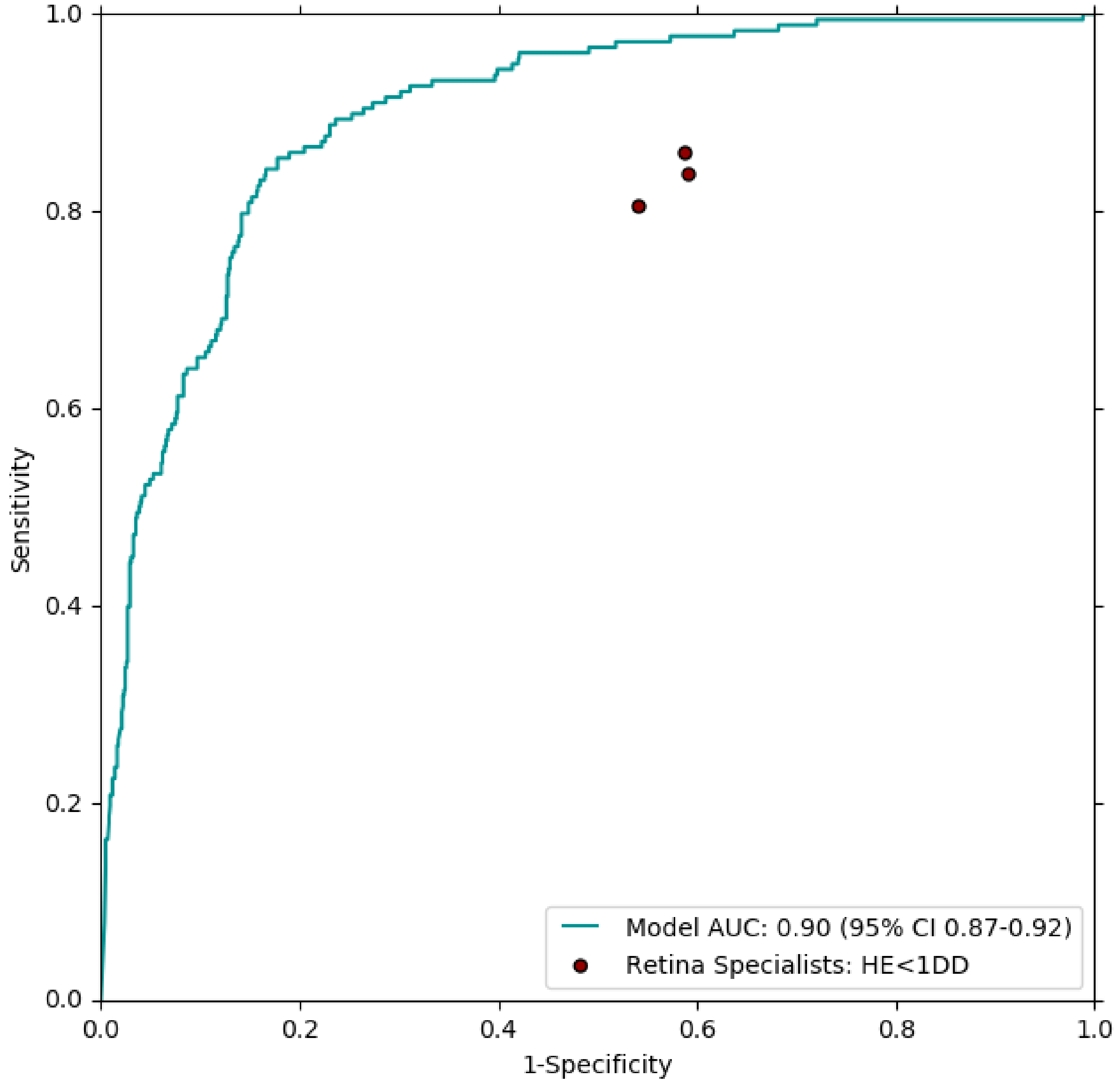

Supplementary Figure 2: Receiver operating characteristic curve of the model with retinal specialists' grades shown as red dots for predicting ci-DME on the primary clinical validation set. All methods (i.e. the model and retinal specialists) rendered their grades using monoscopic fundus images only. The ground truth was derived using OCT at different center point thickness cut-offs for the definition of ci-DME. (A) 280 µm, (B) 300 µm, and (C) 320 µm.

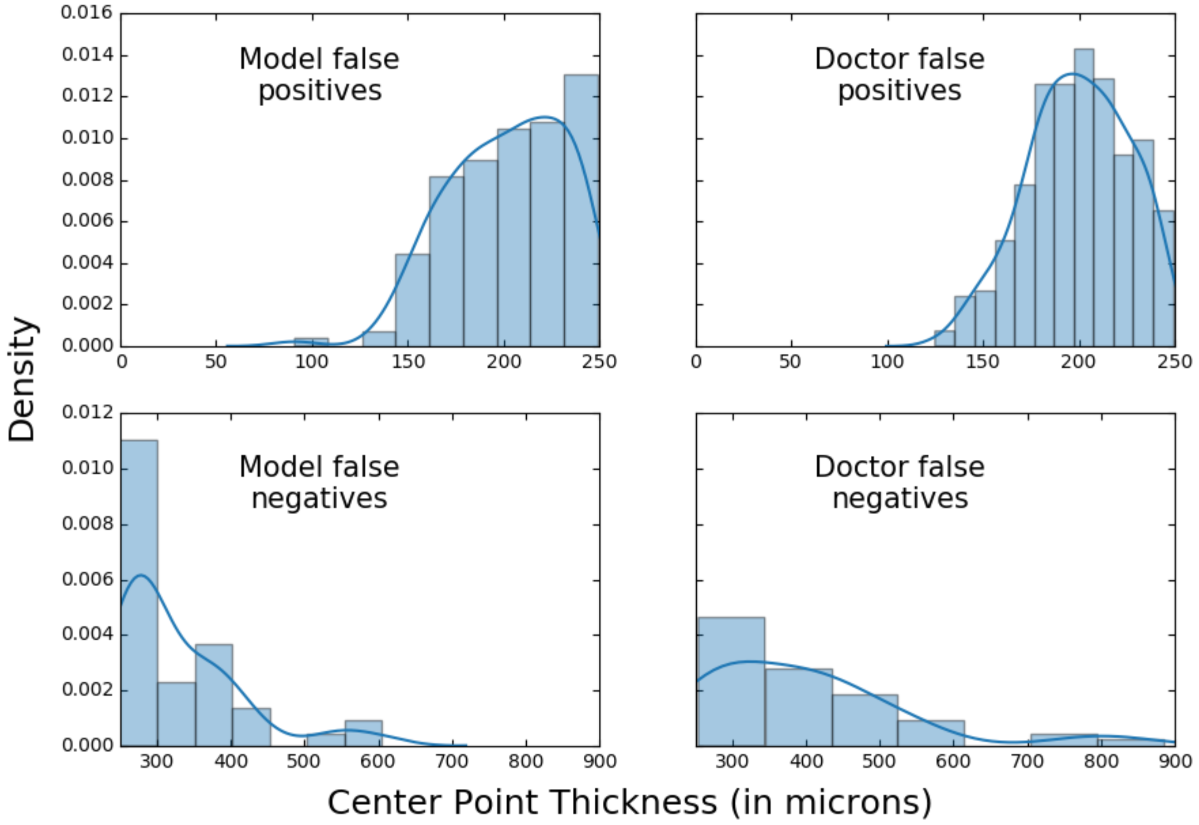

Supplementary Figure 3: Center point thickness distribution of false positive (top) and false negative (bottom) instances for the model (left) and the retina specialists (right). To combine the grades from the three retina specialists we considered the case to be positive for ci-DME only if a majority of retina specialists agreed it was referable for DME. For the model, we chose an operating point that matched the sensitivity of the retina specialists.

Supplementary Tables

Metric	Model	Specialist 1	Specialist 2	Specialist 3
Positive Predictive Value (%), 95% CI	61% [57%-66%]	36% [33%-40%]	37% [33%-41%]	38% [34%-42%]
Negative Predictive Value (%), 95% CI	93% [91%-95%]	88% [85%-92%]	89% [86%-92%]	87% [84%-90%]
Sensitivity (%), 95% CI	85% [81%-89%]	85% [80%-89%]	86% [82%-90%]	81% [76%-86%]
Specificity (%), 95% CI	80% [77%-83%]	44% [40%-47%]	44% [40%-48%]	49% [45%-53%]
Accuracy (%), 95% CI	81% [78%-84%]	55% [52%-58%]	56% [53%-59%]	57% [55%-61%]
Cohen's Kappa, 95% CI	0.58 [0.52-0.63]	0.20 [0.16-0.24]	0.21 [0.17-0.25]	0.22 [0.18-0.27]

Supplementary Table 1: Performance metrics of the model for predicting ci-DME compared with the 3 retinal specialists, calculated only on the images that all 3 retinal specialists deemed gradable (n=948). For the model, we chose an operating point that matched the sensitivity of the retinal specialists to calculate the metrics. Brackets denote 95% confidence intervals.

	Thailand dataset	EyePACS-DME dataset
Patient Population	Patients in Thailand presenting to a retina clinic of a tertiary hospital	Patients in a DR screening program determined based on CFP to have Moderate+ DR
OCT Device	Heidelberg Spectralis	Optovue iVue
ci-DME	Manual measurement of center point thickness >= 250um	Automated measurement of central subfield thickness from Optovue's software >= 300um
Cases with Epimacular Membrane	Excluded	Not excluded
Cases with macular edema from other causes	Excluded	Not excluded
Cases with proliferative DR with neovascular membrane affecting the macula	Excluded	Not excluded
Cases with previous laser treatment	Excluded	Not excluded

Supplementary Table 2: Comparison of the Thailand (training & primary validation) dataset and EyePACS-DME (secondary validation) dataset.

Supplementary Methods

Data augmentation and model hyperparameters

- Inception-v3 network architecture [An implementation is provided at https://github.com/google-research/tf-slim/blob/master/tf_slim/nets/inception_v3.py]].
- Weights of the network are initialized from a checkpoint trained for the Imagenet classification task [Equivalent to the checkpoint available at https://github.com/tensorflow/models/blob/master/research/slim/README.md#pre-trained-models].
- Input image resolution: 587 × 587
- Learning rate: 0.001
- Batch size: 32
- Weight decay: $4 \cdot 10^{-5}$
- Dropout keep probability: 0.8
- An Adam optimizer with $\beta 1$ = 0.9, $\beta 2$ = 0.999, and ε = 0.1 [see `tf.train.AdamOptimizer`]
- Data augmentation (in order):
    1. Random vertical and horizontal reflections [see `tf.image.random_flip_left_right` and `tf.image.random_flip_up_down`]
    2. Random brightness changes (with a max delta of 0.114752799273) [see the TensorFlow function `tf.image.random_brightness`]
    3. Random saturation changes between 0.559727311134 and 1.27488446236 [see `tf.image.random_saturation`]
    4. Random hue changes between -0.0251487996429 and 0.0251487996429 [see `tf.image.random_hue`]
    5. Random contrast changes between 0.999680697918 and 1.77048242092 [see `tf.image.random_contrast`]
- The model was trained for 2 million steps
- Model evaluations performed using a running average of parameters, with a decay factor of 0.9999
