# Supplementary material for: Predicting optical coherence tomography-derived diabetic macular edema grades from fundus photographs using deep learning
Source: Nat Commun. 2020 Jan 8;11:130. doi: 10.1038/s41467-019-13922-8 (PMC6949287; doi:10.1038/s41467-019-13922-8)
Supplement: Supplementary file 2 — Reporting Summary [file 41467_2019_13922_MOESM2_ESM.pdf]

nature research

Corresponding author(s): Lily Peng

Reporting Summary

Nature Research wishes to improve the reproducibility of the work that we publish. This form provides structure for consistency and transparency in reporting. For further information on Nature Research policies, see Authors & Referees and the Editorial Policy Checklist.

Statistical parameters

When statistical analyses are reported, confirm that the following items are present in the relevant location (e.g. figure legend, table legend, main text, or Methods section).

n/a	Confirmed	
☐	☒	The exact sample size (*n*) for each experimental group/condition, given as a discrete number and unit of measurement
☐	☒	An indication of whether measurements were taken from distinct samples or whether the same sample was measured repeatedly
☐	☒	The statistical test(s) used AND whether they are one- or two-sided<br>*Only common tests should be described solely by name; describe more complex techniques in the Methods section.*
☒	☐	A description of all covariates tested
☐	☒	A description of any assumptions or corrections, such as tests of normality and adjustment for multiple comparisons
☐	☒	A full description of the statistics including central tendency (e.g. means) or other basic estimates (e.g. regression coefficient) AND variation (e.g. standard deviation) or associated estimates of uncertainty (e.g. confidence intervals)
☐	☒	For null hypothesis testing, the test statistic (e.g. *F*, *t*, *r*) with confidence intervals, effect sizes, degrees of freedom and *P* value noted<br>*Give P values as exact values whenever suitable.*
☒	☐	For Bayesian analysis, information on the choice of priors and Markov chain Monte Carlo settings
☒	☐	For hierarchical and complex designs, identification of the appropriate level for tests and full reporting of outcomes
☒	☐	Estimates of effect sizes (e.g. Cohen's *d*, Pearson's *r*), indicating how they were calculated
☐	☒	Clearly defined error bars<br>*State explicitly what error bars represent (e.g. SD, SE, CI)*

Our web collection on statistics for biologists may be useful.

Software and code

Policy information about availability of computer code

Data collection	Data collection software used in this study includes that which was provided by fundus camera and OCT equipment manufacturers
Data analysis	Machine learning models were developed and deployed using standard model libraries and scripts in TensorFlow. Custom code was specific to our computing infrastructure and mainly used for data input/output and parallelization across computers.

For manuscripts utilizing custom algorithms or software that are central to the research but not yet described in published literature, software must be made available to editors/reviewers upon request. We strongly encourage code deposition in a community repository (e.g. GitHub). See the Nature Research guidelines for submitting code & software for further information.

Data

Policy information about availability of data

All manuscripts must include a data availability statement. This statement should provide the following information, where applicable:

- Accession codes, unique identifiers, or web links for publicly available datasets
- A list of figures that have associated raw data
- A description of any restrictions on data availability

Restrictions apply to the sharing of patient data that support the findings of this study. This data may be made available to qualified researchers upon ethical approvals from Rajavithi Hospital and EyePACS.

Field-specific reporting

Please select the best fit for your research. If you are not sure, read the appropriate sections before making your selection.

- [x] Life sciences
- [ ] Behavioural & social sciences
- [ ] Ecological, evolutionary & environmental sciences

For a reference copy of the document with all sections, see nature.com/authors/policies/ReportingSummary-flat.pdf

Life sciences study design

All studies must disclose on these points even when the disclosure is negative.

Sample size	Deep learning model accuracy increases with sample size. We included as large a sample for training and validation as possible and also did subsampling experiments to look at the effects of datasize on model performance to show that the model will still benefit from larger training sizes.
Data exclusions	Only cases that were naive to treatment (both intravitreal injections and lasers) were included. Cases where macular lesions may interfere with the diagnosis of DME, such as idiopathic epimacular membrane, macular edema from other causes, or proliferative DR with neovascular membrane affecting the macula, were excluded from analysis.
Replication	A secondary validation set was used to ensure the generalization of the model trained
Randomization	Random test/train splits were generated
Blinding	Test/train splits were automatically generated and sample images were randomly selected.

Reporting for specific materials, systems and methods

Materials & experimental systems

n/a	Involved in the study
[x]	[ ] Unique biological materials
[x]	[ ] Antibodies
[x]	[ ] Eukaryotic cell lines
[x]	[ ] Palaeontology
[x]	[ ] Animals and other organisms
[ ]	[x] Human research participants

Methods

n/a	Involved in the study
[x]	[ ] ChIP-seq
[x]	[ ] Flow cytometry
[x]	[ ] MRI-based neuroimaging

Human research participants

Policy information about studies involving human research participants

Population characteristics	Fundus photos and OCTs were taken from participants with diabetes presenting to eye clinics for care. Most of the patients likely had referrals that resulted in them presenting at the clinic
Recruitment	This is a sample of patients presenting for at eye clinics as a part of routine clinical care
